# Is love right? Prefrontal resting brain asymmetry is related to the affiliation motive

**DOI:** 10.3389/fnhum.2013.00902

**Published:** 2013-12-30

**Authors:** Markus Quirin, Thomas Gruber, Julius Kuhl, Rainer Düsing

**Affiliations:** Institut für Psychologie, University of OsnabrueckOsnabrueck, Germany

**Keywords:** implicit affiliation motive, communion, social motivation, resting EEG alpha asymmetry, VARETA source localization, operant motives test

## Abstract

Previous research on relationships between affective-motivational traits and hemispheric asymmetries in resting frontal alpha band power as measured by electroencephalography (EEG) focused on individual differences in motivational direction (approach vs. withdrawal) or behavioral activation. The present study investigated resting frontal alpha asymmetries in 72 participants as a function of individual differences in the implicit affiliation motive as measured with the operant motive test (OMT) and explored the brain source thereof. Decreased relative right frontal activity as indexed by increased alpha band power was related to low levels of the implicit affiliation motive. No relationships were found for explicit personality measures. Intracranial current density distributions of alpha based on Variable Resolution Electromagnetic Tomography (VARETA) source estimations suggests that the source of cortical alpha distribution is located within the right ventromedial prefrontal cortex (PFC). The present results are discussed with respect to differential roles of the two hemispheres in social motivation.

## INTRODUCTION

### THE AFFILIATION MOTIVE

Affiliation (or communion) constitutes a basic human need considered to have its evolutionary roots in parental care and social relationships of mammals (Bischof, 1976; McClelland, 1987). Although an inherent need in general, strength of the tendency to establish, maintain, or recover affectively positive relationships with other individuals, typically referred to as the affiliation motive, varies across individuals (Atkinson et al., 1954).

Individuals with a high affiliation motive tend to approach situations or people giving reason to expect friendly interactions, i.e., so-called contact incentives typically providing people with positive emotions of love, trust, sympathy, warmth, and security (McClelland, 1987). It has been found that individuals with high levels of the affiliation motive show an increased sensitivity to faces, spend more time calling or writing letters to friends, are liked by others, reach their highest performance in sports when fighting for a team, and make less frequently proposals for group decisions that threaten the cohesion of the group (see Sokolowski and Heckhausen, 2010, for an overview). Notably, US American presidents who had a high affiliation motive according to their inaugural speech, such as Eisenhower, Kennedy, or Nixon, showed increased engagement in equalizing foreign affairs (Winter, 1996, p. 162ff.), which stresses the strong societal relevance of the affiliation motive.

In addition to these behavioral findings, affiliation has also been linked to endocrine processes. Specifically, affiliation contexts such as the formation of social bonds and friendly affection trigger the release of oxytocin (e.g., Carter, 1998) or opioids (Depue and Morrone-Strupinsky, 2005). Moreover, individual differences in the affiliation motive have been linked to increased dopamine levels (McClelland et al., 1987; Sokolowski et al., 1997) and to increased progesterone release in response to affiliation-related stimulation (Schultheiss et al., 2004). This suggests that affiliative processes have specific neural correlates.

Whereas oxytocin and opioids have not directly been related to motives as far as we know, the above-mentioned behavioral findings and endocrine patterns referring to progesterone and dopamine were predicted by indirect motive measures such as the thematic apperception test (TAT; Murray, 1943) or variants thereof (see Schultheiss and Wirth, 2010, for an overview of indirect motive measurement) but not by self-report measures of motives. Because individuals typically have limited conscious access to their motives, the TAT assesses motives implicitly. Specifically, participants are presented with a number of unrelated, ambiguous pictures and are asked to provide story lines that could underlie each picture. Because issues related to one’s personal needs are cognitively highly accessible (Atkinson and McClelland, 1948; Atkinson, 1953; Atkinson and Walker, 1956; McClelland et al., 1980, 1985), they are likely to be weaved in these stories, constituting a basis for scoring the test later on. In particular, it is broadly assumed and empirically confirmed that implicitly measured motives are more powerful than self-report motive measures in predicting spontaneous behavior as compared to planned and consciously controlled behavior (McClelland et al., 1989). Therefore, implicit motives assessment also qualifies as an appropriate predictor of physiological processes (Schultheiss and Wirth, 2010).

### HEMISPHERE ASYMMETRY IN MOTIVATION

Previous research investigating relationships between motivation and hemisphere asymmetry concentrated on motivational direction (Harmon-Jones et al., 2010). Broadly, two directions of motivational striving can be distinguished, approach motivation versus avoidance motivation (Lewin, 1936; Gray and McNaughton, 2000; Elliot and Thrash, 2002). Whereas approach motivation drives us to draw near to rewarding stimuli, situations or persons, avoidance motivation drives us to withdraw from threats or otherwise unpleasant stimuli, situations, or persons. Consequently, approach motivation is generally linked to positive emotions, whereas avoidance motivation is generally linked to negative emotions. However, an exception refers to anger, which is negative but still an approach-related emotion (Harmon-Jones et al., 2010).

According to a popular model, approach motivation is linked to relatively higher activity at left as compared to right prefrontal electrodes putting forward the assumption that the left PFC is involved in approach motivation, whereas the right PFC is involved in withdrawal motivation (e.g., Davidson, 1992; van Honk and Schutter, 2006; Harmon-Jones et al., 2010). In this research, alpha power has typically been used as an inverse marker of cortical activity in a way that lower levels of alpha power indicate higher levels of activity (e.g., Cook et al., 1998). The motivational direction model of frontal hemisphere asymmetry saw a particular boost from research on the negative emotion anger, suggesting that relative left frontal activity is related to the approach aspect rather than to the valence aspect of an emotion (Harmon-Jones et al., 2010). According to a contrasting model, behavioral activation versus inhibition rather than motivational direction is the crucial condition for lateralization. As such, avoidance motivation in terms of fearful flight, which refers to behavioral activation, was found to be associated with relative left rather than right hemispheric activation (Wacker et al., 2008).

Central to the present work, many studies have established that frontal asymmetry is not only related to motivational states but also to motivational traits (de Pascalis, 1993; Hagemann et al., 1999; Coan and Allen, 2003b; Hewig et al., 2006). In this research, dispositional approach motivation was indicated by individual differences in behavioral activation, extraversion, or positive affectivity, whereas dispositional avoidance motivation was indicated by individual differences in behavioral inhibition, neuroticism, or negative affectivity (see Elliot and Thrash, 2002, for a link between these concepts). Not least, such findings are compatible with evidence suggesting that frontal asymmetry constitutes a relatively stable marker of individual differences (Tomarken et al., 1992).

However, although many studies suggest relative left versus right frontal preponderance for approach versus avoidance related motivational traits such as behavioral activation versus inhibition (Coan and Allen, 2003a; Wacker et al., 2008), respectively, the amount of absent or even contrary findings is not negligible (e.g., Hewig et al., 2006; Wacker et al., 2010, for a meta-analysis). Wacker et al. (2010), for example, analyzed the results of 26 published articles and dissertations measuring frontal alpha asymmetry and individual differences in trait approach motivation such as behavioral activation system (BAS) or agentic extraversion. Most of the analyzed studies showed no significant relationship between trait approach motivation and left frontal activity or even showed inverse relationships (e.g., Hayden et al., 2008). In fact, the overall mean weighted effect size was relatively small (*r* = .047). This led the authors to conclude that if an association between alpha asymmetry and behavioral activation exists, it is too weak and too inconsistent to be of much interest. Not least, the results also clearly indicated a systematic publication bias in favor of significant results. As suggested by the authors, future research is needed that focuses on factors potentially explaining the inconsistency in the relationships found between trait motivation and frontal asymmetry. In the following paragraphs, we emphasize three potential reasons for this inconsistency.

First, previous research investigating hemisphere asymmetries in motivation did not account for the particular social need, e.g., the affiliation motive (or the power motive) that underlies approach motivation. This omission is nicely exemplified by a problem associated with the construct of extraversion, a trait closely linked to approach motivation (e.g., Elliot and Thrash, 2002). As stressed by Depue and Morrone-Strupinsky (2005), extraversion confounds the two dimensions of affiliation and agency (“power”), and, congruently, measures of extraversion are positively correlated with measures of both, motives for affiliation and for power (e.g., Engeser and Langens, 2010). In fact, previous research on relationships between extraversion and hemispheric asymmetries led to inconsistent findings (e.g., Kline, 1999), as it was also the case for behavioral activation, which is closely related to extraversion (Wacker et al., 2010). Accordingly, it appears to be promising to examine brain activity as a function of the social motivational subdimensions of affiliation and power rather than solely to broad personality dimensions related to the more general concept of approach motivation.

A second reason for inconsistent results might be the fact that responses in self-report motives questionnaires are typically biased by a number of factors such as social desirability, self-deception, or a lack of introspective abilities. By contrast, implicit tests measure motives via spontaneous responses to motive-relevant stimuli rather than via attitudes toward the own person (McClelland et al., 1989). Therefore, using a measure that assesses the social content of a motivational trait, i.e., affiliation or power, by circumventing self-reports of participants appears to be promising.

Third and last, previous research on frontal asymmetries in motivational processes, trait or state, did not investigate the brain source of asymmetries. An exception is a study by Pizzagalli et al. (2005) who reported left dorsolateral PFC activity underlying approach motivation in response to monetary reward. However, these findings refer to state rather than to trait approach motivation. In fact, it is well-known that the source of a signal measured at an electrode site is not necessarily located in close proximity but can originate from remote or even contralateral areas (Luck, 2005). Therefore, source reconstructions of signals measured in electrode space are necessary to make a statement about the brain areas that are actually involved in motivational processes.

### AFFILIATION MOTIVE AND HEMISPHERE ASYMMETRY

Although the affiliation motive has proved to be a valid predictor of spontaneous behavior and neuroendocrine processes, direct evidence on differential hemisphere involvement in the affiliation motive is scarce to date. Nevertheless, research on prosocial processes, which compose major constituents of the affiliation motive (McClelland, 1987; see also Seyfarth and Cheney, 2013) and which individuals of high levels in the affiliation motive are thus inclined to show more often (e.g., McAdams and Constantian, 1983), suggests a right hemisphere advantage for the affiliation motive. For example, empathy has been related to the right posterior cortex (Adolphs et al., 2000; Decety and Lamm, 2007) and the right PFC (Shamay-Tsoory et al., 2003; Tullett et al., 2012). Similarly, the right PFC has been found to be linked to the attribution of others’ mental states (Platek et al., 2004) and to cooperation (Knoch et al., 2006). Moreover, patients with lesions in the right ventromedial PFC showed impaired mental states attribution (Shamay-Tsoory et al., 2005) or even met criteria for acquired sociopathy (Tranel et al., 2002), suggesting a particular role of this region in affiliative processes.

Recent visual field studies more directly suggest right-lateralized asymmetry for affiliation. In a study from Mohr et al. (2008), for example, participants made lexical decisions on positive and negative affiliation-related words in a bilateral simultaneous presentation paradigm. The authors found that positive affiliation-related words presented in the left visual field (right hemispheric advantage) were processed faster and more accurately than negative affiliation-related words. Such differences were not found for right visual field (left hemisphere) presentations. In a series of studies, Kuhl and Kazén (2008) used a modified version of the dot-probe task (MacLeod et al., 1986) to investigate attentional biases occurring after the presentation of pictures or words related to the affiliation motive. Affiliation and power stimuli were either approach-related (need satisfaction) or avoidance-related (need frustration). The authors found an attentional bias toward affiliation-related pictures or words if presented in the left visual field (right-hemisphere advantage) as compared to the right visual field. Notably, those effects were independent of the approach or avoidance related content of the motive-related stimuli, which suggests that affiliation may be lateralized independent of motivational direction. Additionally, the authors found that individuals with high scores on affiliation motives, respectively, showed stronger effects than those with low scores, suggesting the role of trait-like affiliation motivation. However, the method used in these studies as well as in the study from Mohr et al. (2008) is based on a behavioral paradigm (visual field presentations). Therefore, using physiological methods such as EEG is necessary to substantiate these findings.

### PRESENT RESEARCH AND HYPOTHESES

The present study focuses on relationships between individual differences in the affiliation motive and cortical asymmetries using laterality indices in the EEG alpha frequency band and explores the brain sources for potential asymmetries. Because motivation and emotion has been linked to alpha asymmetries at frontal electrodes (Hagemann et al., 2002; Coan and Allen, 2003a; Davidson, 2003; Harmon-Jones, 2007), our hypotheses target this region. Specifically, we expect that relative right frontal activity (or reduced activity) is related to high levels (or low levels) of the affiliation motive. In particular, we hypothesize to find this relationship for the implicit rather than the explicit affiliation motive. Further, we explore whether potential effects of the affiliation motive are independent of the power motive, another important trait related to approach motivation. Likewise, we control effects of trait behavioral activation versus behavioral inhibition (Gray and McNaughton, 2000) or trait anger, i.e., personality traits related to approach versus avoidance motivation that have previously been found to be linked to frontal asymmetry.

Due to the fact that this study is one of the first attempts to identify neural substantiations of the affiliation motive, we also explored electrode sites other than the frontal regions. Further, we used a distributed source model to estimate primary current density distributions of alpha band activity to identify brain regions likely responsible for establishing the correlational pattern found in electrode space.

## MATERIALS AND METHODS

### PARTICIPANTS AND PROCEDURE

Seventy-two right-handed students (32 female), aged 18–33 (*M* = 22.8, SD = 3.1), were recruited at the University of Osnabrueck via flyers and postings. The study was approved by the local Ethics Committee. The students were informed about the EEG procedure and gave written consent to participate. At the end of the experiment, participants received 20 Euro (about $25) in return for their participation.

In a first session participants filled out a battery of measures that included tests for the assessment of individual differences in affiliation (relationship) and power (dominance) motives. Individual appointments were made for a second session taking place about 4 weeks later, in which resting EEG was recorded while the participant sat in a comfortable chair. EEG was recorded in occasions of eight 1 min resting periods, where four occasions were recorded with eyes open and four with eyes closed. The measurements were counterbalanced across participants according to one of two sequences of eyes open (O) and eyes closed (C) conditions (O-C-C-O-C-O-O-C or C-O-O-C-O-C-C-O). Participants were informed via a recorded voice when to open or close their eyes.

#### EEG assessment

Electroencephalography was recorded with a stretchable electrocap (Brain Cap; brand Easy Cap). 29 electrodes were placed according to the extended 10–20% system based on Jasper (1958), referenced to the vertex. FCZ was used as an integrated ground. Additionally, Electro-oculogram was recorded to control for artifacts due to eye movements. After the scalp under the electrodes had been cleaned with alcohol, an abrasive mild gel was used to reduce impedances. All electrode-impedances were below 5 kΩ and homologous sites were within 1 kΩ of each other. EEG was recorded with the Brain Amp Standard (brand Brain Products GmbH). The sampling rate was set to 500 Hz.

All off-line procedures were conducted using EEGLAB (Delorme and Makeig, 2004). EEG raw data was re-referenced to TP9 and TP10 mastoid electrodes. Artifacts due to eye-movements and blinks were reduced by a blind source separation algorithm (Gómez-Herrero, 2007). Artifact-reduced 1 min epochs were segmented in periods of 2 s and were extracted through a Hamming Window. Consecutive epochs had a 75% overlap to minimize data loss due to windowing. Epochs were automatically rejected if the amplitude at one sample exceeded ±75 μV. For remaining epochs a 30 Hz low-pass filter was applied. Resulting data were zero padded and submitted to a fast Fourier transformation with a resolution of 0.48 Hz. Power values within the alpha band (8–13 Hz) were averaged across all epochs. Power values were natural-log-transformed (ln) to obtain normalized values (Allen et al., 2004).

Asymmetry indices for each 1 min period were calculated by subtracting ln alpha frequency of left electrode sites from ln alpha frequency of homologous sites of the right hemisphere (e.g., F8-F7, F4-F3) with higher scores indicating a relatively stronger left-sided activation. Following previous literature (e.g., Coan and Allen, 2003b, for a review), only frontal electrode pairs (i.e., FP2–FP1, F4–F3, and F8–F7) were analyzed in a first step. Cronbach’s alpha coefficients of the asymmetry indices for homologous electrode sites from the eight time periods ranged from 0.82 to 0.94. Therefore, we computed correlations between personality variables with the average alpha asymmetry scores.

To identify brain regions underlying significant asymmetries we conducted a source localization analysis using VARETA (Bosch-Bayard et al., 2001). This method provides a spatial intracranial distribution of primary current densities in source space, which is best compatible with the amplitude distribution found in electrode space (cf. Gruber et al., 2006). The complex Fourier coefficients of the frequency bins relating to the 8–13 Hz band were first averaged and subsequently submitted to the inverse solution algorithm. As possible sources of the signal 3244 voxels (grid points) of a 3D grid of 7 mm grid spacing were used. This grid arrangement was placed in registration with the probabilistic brain tissue maps available from the Montreal Neurological Institute (MNI, Evans et al., 1993). MNI coordinates were converted into Talairach coordinates using the Yale non-linear converter (; Lacadie et al., 2008). Because primary current density scores show a great interindividual variability they were normalized.^1^ Because each voxel contains three direction information of the primary current density, the maximum value within each voxel was chosen to calculate correlations with psychological measures.

#### Psychological measures

**FIGURE 1:**
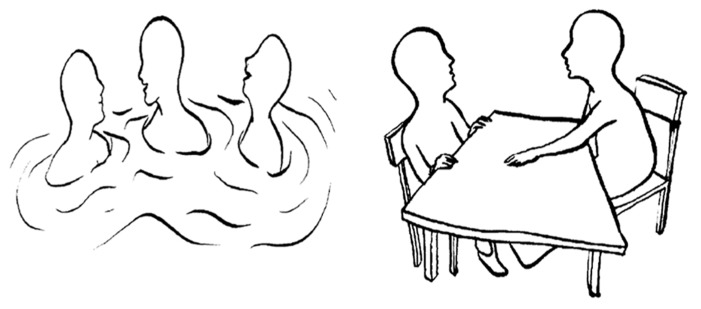
**Two example pictures from the operant motives test**.

To assess the affiliation motive we applied the OMT (Kuhl and Scheffer, 1999), which is similar to the TAT or the Picture Story Exercise (McClelland et al., 1989) in that participants interpret how people feel or think in a set of pictures, which provides the basis for later motive scoring by an expert rater. In the OMT, however, participants are provided with 15 schematic drawings of social interactions each depicting one or more characters (see **Figure 1**, for two example pictures). Participants are instructed to choose one of the characters and – in contrast to the TAT – to write down in short-form (e.g., using keywords) their spontaneous associations to the following four questions: (1) “What is important for the person in this situation and what is the person doing?,” (2) “How does the person feel?,” (3) “Why does the person feel this way?,” and (4) “How does the story end?” Pictures that contain an approach-related affiliation (power) theme score a point on the affiliation (power) scale from 0 to 15. OMT scoring was carried out independently by two well-trained assistants who have (a) previously coded more than a thousand OMTs, (b) received continuous feedback concerning their agreement with expert ratings over a period of 3 years, and (c) reached an average inter-rater agreement above 0.90. Evidence confirming the validity of the OMT has been re ported by a number of studies (Hofer et al., 2008; Quirin et al., 2009)^2^.

To control for individual differences in the sensitivity of the BAS and the behavioral inhibition system (BIS; Gray, 1987), we applied corresponding scales from Carver and White (1994; German version by Hartig and Moosbrugger, 2003). Fifty-eight items measure the three dimensions of trait anxiety and frustration (BIS), drive and pleasure (BAS), as well as anger. The scales show appropriate reliabilities (Cronbach’s alphas between 0.79 and 0.85) and an adequate factor pattern (Hartig and Moosbrugger, 2003).

## RESULTS

First, we computed correlations between social motives and more general motivational traits of BIS and BAS. As depicted by Table 1, the affiliation motive was inversely related to the power motive. Moreover, the affiliation motive was positively correlated with BAS. By contrast, the power motivation was negatively correlated with BAS. Because the two social motives were inversely correlated with each other, we computed a multiple regression analysis to explore whether one of the two motives was more closely related to BAS than the other. Certainly neither of the two motives reached significance after controlling for the respective other motive, *p*s > 0.18. Consistent with the findings from the study which validated the scales (Hartig and Moosbrugger, 2003), trait anger was positively associated with BIS but was unrelated to BAS.

**Table 1:** Means, SD, and correlations for affiliation and power motives, BIS, BAS, and trait anger with resting EEG alpha power asymmetry scores.

	II	III	IV	V	VI	VII	M	SD
Affiliation motive (I)	-0.58^*^^*^	-0.03	0.27^*^	-0.01	-0.26^*^	-0.24^*^	2.14	1.23
Power motive (II)		0.09	-0.24^*^	0.09	0.16	0.13	7.46	2.34
Behavioral inhibition (III)			-0.27^*^	.46^*^^*^	0.10	0.11	2.49	0.49
Behavioral activation (IV)				-0.02	-0.20	0.06	2.91	0.47
Trait anger (V)					-0.19	0.15	2.31	0.53
^+^Asymmetry (F4-F3; VI)						0.26^*^	0.03	0.22
^+^Asymmetry (T8-T7; VII)							0.02	0.28

N = 72; ^*^p < .05; ^**^p < .01 (two-tailed).

*^+^Negative correlations indicate that (low) scores in psychological measures are associated with (low) relative right hemispheric activity.*

**FIGURE 2:**
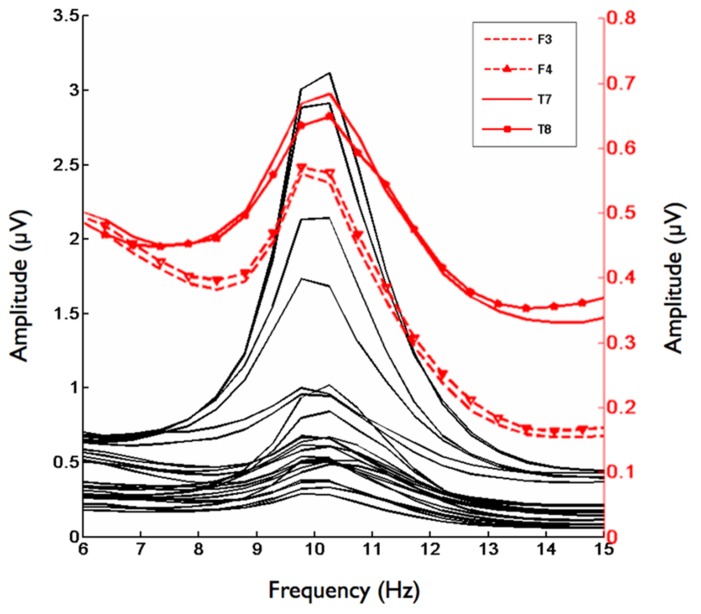
**Grand mean for the frequency range from 6–15 Hz for 29 Electrodes**. Red lines indicate homologous electrode sites at which asymmetry scores significantly correlated with the implicit affiliation motive. Black lines indicate remaining electrode sites.

**FIGURE 3:**
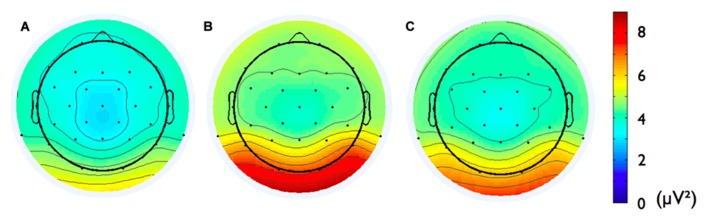
**Topographic distribution of the alpha frequency spectrum (8–13 Hz) for 8 min resting state EEG for (A) eyes-open condition, (B) eyes-closed condition, and (C) the two conditions averaged**.

Second, we analyzed the alpha band power spectrum by averaging across all participants and epochs. As can be seen in **Figure 2**, we found a peak at 10 Hz suggesting that the frequency range between 8 and 13 Hz, which has traditionally been selected to analyze frontal alpha band asymmetries (cf. Allen et al., 2004), appears to be an appropriate marker of brain activity in the present sample. **Figure 3** depicts the topographical distribution of alpha power separately for eyes open versus eyes closed. As expected, alpha band suppression was higher in the eyes-open condition. Furthermore, alpha power was highest for occipital and parietal electrode sites, which corresponds with the pattern typically reported in the literature (e.g., Nunez et al., 2001).

Most central to our study was the analysis of the affiliation motive as well as of other motivational traits with alpha power asymmetry scores at frontal electrode sites. As depicted by Table 1, the implicit power motive, the explicit power, and affiliation motives, as well as BIS and BAS were uncorrelated with frontal asymmetry. By contrast and in line with our hypothesis, the implicit affiliation motive showed a significant negative correlation with frontal asymmetry at F4–F3, *r* = -0.26, *p* = 0.03. To test for statistical robustness of the result, we applied a bias-corrected and accelerated (BCa) bootstrapping method on the basis of 1000 samples. The 95% confidence interval [-0.032 -0.45] did not cross zero (SE = 0.103; bias = 0.007), which suggests that this correlation is not driven by potential outliers.

**FIGURE 4:**
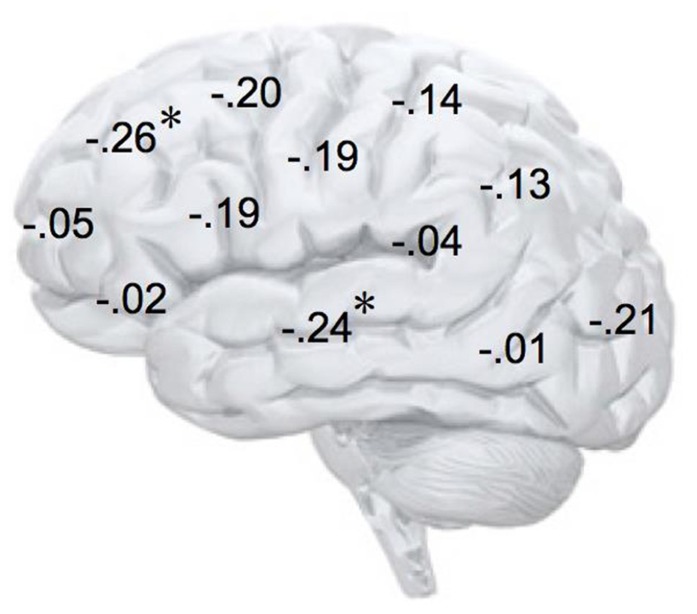
**Correlation coefficients of the relationship between alpha asymmetry scores at each electrode site and the implicit affiliation motive**. *N* = 72; ^*^p < 0.05; negative coefficients indicate lower relative right-lateralized activity being associated with low levels of implicit affiliation.

**FIGURE 5:**
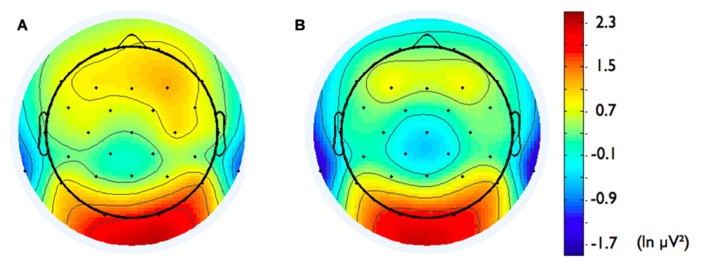
**Topographical distribution of ln alpha power for participants with (A) low affiliation motive (below percentile 25) and **(B)** high affiliation motive (above percentile 75)**.

In addition, we explored relationships between motivational traits and asymmetry scores at non-frontal scalp sites. None of the correlations were significant for the power motive, BIS, BAS, and anger (not depicted). By contrast, as shown by **Figure 4**, the affiliation motive showed a significant negative correlation with one temporal asymmetry score (T8-T7), *r* = -0.24, *p* = 0.04, CI of BCa bootstrapping [-0.025 -0.43], SE = 0.115, bias = -0.002. To illustrate the relationship between affiliation motive and ln alpha power distribution, **Figure 5** depicts topographical plots separately for participants scoring low (below percentile 25) versus high (above percentile 75) on the implicit affiliation motive. The graphs illustrate that low relative right frontal activity is associated with low levels of implicit affiliation but that little or no asymmetry exists for individuals with high levels of implicit affiliation.

**FIGURE 6:**
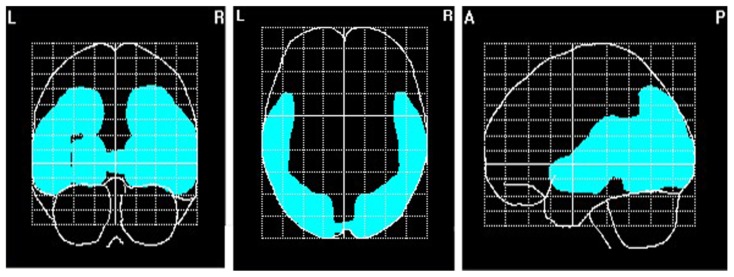
**Source localization of EEG alpha (8–13 Hz)**. *N* = 70; marked voxels indicate primary current density values for the alpha frequency spectrum 1 SD above the mean.

**FIGURE 7:**
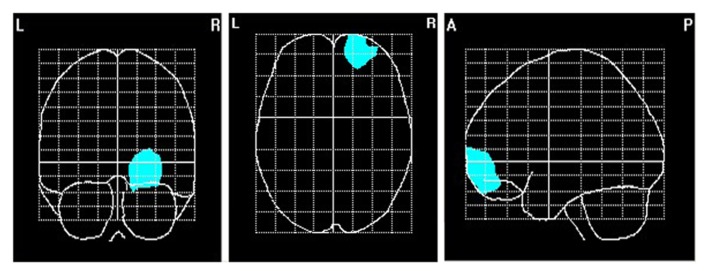
**Area of primary current density values of alpha correlating significantly with the affiliation motive**. *N* = 70; voxels within the right ventromedial PFC that were significant at *p* < 0.05.

To explore brain regions responsible for the significant correlations with the affiliation motive in electrode space, we conducted VARETA source localization. To check for validity of the solution at first, we inspected the primary current density scores of the alpha band. Two participants were excluded from the analysis due to severe distortions in the source space solution without substantial changes of correlation coefficients at scalp sites. **Figure 6** depicts all voxels with primary current density scores one standard deviation above the mean. As expected and in line with topographical distributions described by Nunez et al. (2001) higher values were found for occipital, parietal, and temporal areas. Moreover, the estimated sources of alpha were also similar and thus consistent with the reported topographical distribution at scalp sites. In a next step, we correlated primary current density values from the VARETA solution with affiliation scores. As can be seen in **Figure 7**, we found significant activation in the right ventromedial PFC (Brodmann Area 10) with a center of gravity at MNI coordinates *x* = 21, *y* = 54, *z* = -2 and Talairach coordinates *x* = 20, *y* = 49, *z* = -2 which was correlated with affiliation scores in the expected direction, *r* = -0.25, *p* = 0.03, CI of BCa bootstrapping [-0.084 -0.400], SE = 0.08, bias = -0.003. No other area of significant voxels could be identified.

## DISCUSSION

This study investigated relationships of the affiliation motive with asymmetric distribution of resting frontal EEG alpha band power. We also determined cortical generators of this relationship using a distributed source model. As expected, we found a relationship between the affiliation motive and relative right frontal activity at scalp sites. Specifically, participants scoring low on implicit affiliation showed decreased activation over the right PFC, which could be attributed to decreased activity in the right ventromedial PFC. The present findings are compatible with recent evidence on the involvement of the right hemisphere in affiliation motivation, as found by accelerated stimulus reactions after affiliation primes presented in the left compared to the right visual field (Kuhl and Kazén, 2008; see also Mohr et al., 2008). Our neurophysiological results extend these findings with respect to the affiliation motive by specifying the brain region associated with trait affiliation.

The ventromedial PFC along with the adjacent orbitofrontal cortex constitute PFC areas most strongly involved in emotion and motivation (Olsson and Ochsner, 2008). The ventromedial PFC has been found to play a major role in intuitive judgment and decision making (Bechara et al., 1999), emotion regulation (Kalisch et al., 2006; Olsson and Ochsner, 2008), attention and adherence to social knowledge (Blair and Cipolotti, 2000), representing internal mental states (Christoff and Gabrieli, 2000), as well as in mentalizing about self and similar others (Amodio and Frith, 2006; Gilbert et al., 2006; Olsson and Ochsner, 2008).

Evidence on functional laterality in ventromedial PFC predominantly derives from lesion studies. Specifically, patients with lesions of the right but not left ventromedial PFC suffer profound deficits in empathy (Shamay-Tsoory et al., 2003) and affective components of theory of mind (Shamay-Tsoory et al., 2005) as well as in social and interpersonal behavior resembling that of sociopaths (Tranel et al., 2002). This pattern of results is in line with recent findings of relative right prefrontal activity (reduced alpha) being associated with empathic reactions (Tullett et al., 2012). Moreover, lesion studies suggest a role of the right but not left ventromedial PFC in intuitive decision-making (Tranel et al., 2002) and creativity (for reviews, see Mihov et al., 2010; Shamay-Tsoory et al., 2011). This is compatible with evidence on a link between affiliation motive and intuitive thought as assessed via correct identification of remote associates (Kuhl and Kazén, 2008; Quirin et al., 2013a). Because of the likely existence of relationships between affiliation motive and empathic abilities or intuitive processing, it cannot be excluded that the present neural data might be influenced by individual differences in empathy or intuitive processing, respectively, which were not assessed here. Nevertheless, because empathy and intuitive processing are assumed to be components of the affiliation motive (Kuhl and Koole, 2008), our work strengthens evidence from lesion studies on social functions of the right ventromedial PFC by adding functional brain data.

Previous research found that relative activity (reduced alpha) at left prefrontal scalp electrodes is linked to state and trait levels of approach motivation (Harmon-Jones et al., 2010). However, concerning trait motivation in particular, the literature is very inconsistent (e.g., Wacker et al., 2010). One reason for this inconsistency might be seen in the fact that previous research on hemisphere asymmetries did not control for whether approach motivation was driven by affiliation or other motive concerns such as power, sex, or achievement (but see Quirin et al., 2013b). In fact, at least some research on frontal asymmetry might be biased to favor power motivation as a particular type of approach motivation. Specifically, state and trait measures of positive affect or approach motivation such as the positive affect scale of the Positive and Negative Affect Schedule (Watson et al., 1988) or the Behavioral Activation Scale (Carver and White, 1994) assess the degree to which individuals experience positive emotions associated with dominance (e.g., determined, strong, proud) rather than affiliation. Consequently, it is possible that frontal asymmetries in emotional processing are due to differences in the dimension of power rather than to approach motivation in general.

Previous research on the affiliation motive found beneficial effects of a high affiliation motive on cardiovascular stress reduction (McClelland, 1979) or on immune system functioning (Jemmott, 1982). This might be attributed to the stress-reducing effects of progesterone, which has been found to be elevated in individuals with higher levels of affiliation motive (Wirth and Schultheiss, 2006). Since affiliation processes, although not yet the affiliation motive itself, have been linked to increased oxytocin (Carter, 1998) and opioid involvement (Depue and Morrone-Strupinsky, 2005), these neurotransmitters might play an additional role here (see also Schweiger et al., 2013). Notably, much evidence suggests a role of the ventromedial PFC in stress and negative affect regulation (Ochsner and Gross, 2005; Kalisch et al., 2006; Urry et al., 2006; Cooney et al., 2007). This is in line with our findings and suggests that activity of the right ventromedial PFC might constitute a further variable mediating stress buffering effects of the affiliation motive. Compatible with this view but speculatively, the present findings may also be interpreted in the following way: since affiliation is associated with both a broadening of attention (Kuhl and Kazén, 2008; Quirin et al., 2013a) and with oxytocin and opioid elicitation, phenomena both being linked to the consummatory (“liking”) rather than the anticipatory (“wanting”) phase of goal attainment, it is likely that trait affiliation and right PFC activity are linked to positive emotions of relaxation and enjoyment rather than to agentic, approach-related emotions like appetence or anger. However, future research is needed to corroborate this assumption.

The present study investigated resting frontal asymmetry and motivational traits. However, there is also evidence that resting frontal asymmetry might be influenced by situation variance rather than by trait person variance (e.g., Coan and Allen, 2003b, 2004; Gable and Harmon-Jones, 2008). Thus, it cannot be excluded that relative right frontal asymmetry originating in the right ventromedial PFC may refer to state rather than to trait activity among participants with high levels of affiliation motive. However, considering that we measured under baseline conditions, the likelihood that motivational states were induced should be low. As such, potential unintended inductions of affiliation states in a few individuals, unless systematic, should not have contributed strongly to the present effect.

One might argue that the spatial sampling of the scalp with 29 electrodes is not high enough to reconstruct robust generators of the EEG signal in the brain. For instance, Michel et al. (2004a), b claimed that at least 60 sensors are needed to correctly sample the scalp electric field submitted to the source localization procedure. However, in many studies regarding the precision of source reconstructions, test dipolar sources are used. Dipolar sources correspond to highly focal activity. Obviously, in these cases the localization error is sensitive to electrode numbers (since information about variations of the voltage distribution might be lost due to spatial undersampling). However, in the present study we estimated sources of EEG alpha rhythm, which is very well established to be generated by a widely distributed network (e.g., Nunez, 2000; Nunez et al., 2001). Furthermore, we did not rely on a dipolar but a distributed source model. Both facts speak against a substantial informational gain by adding more electrodes. For instance, Trujillo-Barreto et al. (2004) demonstrated that the localization error of cLORETA – an approach very similar to VARETA – is not too much affected by dramatic changes in the number of electrodes (from 19 to 128). In cases of widespread activity generating a spatially smooth topography (cf. **Figure 3**), adding more electrodes does not add ample new information. This holds under the premise that the electrodes are equally distributed across the scalp, which was the case in our study. Thus, we have good reasons to assume that the present source reconstructions are valid. This assumption is underpinned by our estimation of alpha activity sources *per se* (see **Figure 6**), which is well in line with the literature (e.g., Manshanden et al., 2002; Laufs et al., 2003; Hoogenboom et al., 2006; Tuladhar et al., 2007).

The implicit affiliation motive was weakly correlated with behavioral activation. This is in line with the notion that the affiliation motive, as measured here, refers to approach motivation (e.g., Gable, 2006) and that the affiliation motive in general is confounded with extraversion (Depue and Morrone-Strupinsky, 2005), a construct that is also related to approach motivation Elliot and Thrash, 2002). However, previous research found no significant correlations between implicit affiliation motive and behavioral activation (Pang and Schultheiss, 2005) or extraversion (Winter et al., 1998; Engeser and Langens, 2010). However, these authors used alternative measures of implicit affiliation and future research is needed to investigate the relationships between implicit motives and explicit personality traits more systematically by simultaneously applying various implicit motive measures, which typically differ in their properties.

## CONCLUSION

The present research pioneered in uncovering the neural correlates of the affiliation motive using EEG. Compatible with behavioral studies on a right hemispheric involvement, we found low scores on the affiliation motive being related to decreased relative right frontal activity, which had its source in reduced activity within the right ventromedial PFC. Our results put forward a more differentiated view on hemispheric lateralization of approach motivational traits with affiliation potentially being special in that it appears to be related to right PFC processing.

## Conflict of Interest Statement

The authors declare that the research was conducted in the absence of any commercial or financial relationships that could be construed as a potential conflict of interest.

## Footnotes

1 Normalization of primary current density scores was calculated as follows: N ij = ( W ij - M inj)/(Maxj - Minj) where N equals a normalized primary current density for the j th person in the i th voxel, W ij the initial value,M axj the maximum and M inj the minimum of the j th person.

2 We additionally assessed explicit affiliation and power motives by the Personal Values Questionnaire (McClelland, 1991) but the findings were far from being significant. This result is in line with our argument outlined in the introduction. We do not report on these findings in order to keep our focus on implicit motives and thus to keep the manuscript less complex.
